# Hyperreactio Luteinalis: A Rare Phenomenon Complicating Gestational Trophoblastic Disease Presenting as Acute Abdomen in the Convalescence Period

**DOI:** 10.7759/cureus.55382

**Published:** 2024-03-02

**Authors:** Sachin Rathod, Neema Acharya, Amardeep Shanoo, Preeti Mishra, Anubha Dande

**Affiliations:** 1 Department of Obstetrics and Gynaecology, Datta Meghe Institute of Higher Education and Research, Wardha, IND; 2 Department of Pathology, Datta Meghe Institute of Higher Education and Research, Wardha, IND

**Keywords:** emergency laparoscopy, reproductive health, cyst rupture, molar pregnancy, theca lutein cyst

## Abstract

The hydatidiform mole is a rare gynaecological condition originating from trophoblastic cells, with an incidence of 1-3 per 1000 pregnancies. Theca lutein cysts (TLCs) and an invasive mole are rarely observed in association with a partial mole. This case describes an unusual case involving a 17-year-old primigravida at 11 weeks of gestation. She presented with abdominal pain and was diagnosed with a molar pregnancy with post-evacuation rupture of TLC, presenting as an acute abdomen, subsequently undergoing laparoscopy. Post-molar pregnancies exhibit a highly variable course, ranging from recurrent pregnancy loss and stillbirths to preterm deliveries and recurrent molar pregnancies. Few studies are available on obstetric outcomes after a molar pregnancy; most available data originate from national databases and monocentric research.

## Introduction

The term "gestational trophoblastic disease" (GTD) refers to a diverse collection of uncommon tumours that are characterized by the irregular growth of trophoblastic tissue. The most prevalent GTDs are partial and complete molar pregnancies. Although they typically count as benign conditions, they have the potential to progress into premalignant conditions such as gestational trophoblastic neoplasia. Three types of functional cysts in the ovary occur during pregnancy: corpus luteal, theca lutein, and follicular. Theca lutein cysts (TLCs) are the least common among them [1]. TLCs during pregnancy are usually not associated with any symptoms, are self-limiting, and are discovered incidentally by ultrasonography or during caesarean section. The association of TLCs with a partial mole is rare [2].

Hyperreactio luteinalis (HL) has an unclear aetiology. However, it is believed that high human chorionic gonadotropin (hCG) levels and heightened ovarian sensitivity to its long exposure might manifest as a severe ovarian reaction that results in the development of TLCs. It is extremely rare in pregnancies unrelated to trophoblastic disease [3]. Post-molar pregnancies exhibit a highly variable course. Our case involves an unusual incidence of TLCs in a singleton pregnancy, presenting as a post-evacuation acute abdomen due to cyst rupture, requiring emergency laparoscopic exploration.

## Case presentation

A 17-year-old female presented to the outpatient department due to the acute onset of abdominal pain that had been worsening every day for the previous six days. The pain originated in the lower abdomen as a stretching sensation and then spread throughout the entire abdominal area. The pain was dull, aching, and progressive in nature. On the pain scale, she rated it as seven out of 10, with no aggravating or alleviating factors for her discomfort, accompanied by four to five episodes of nausea and vomiting. Her last menstrual period was three months ago. Prior to that, her menstrual cycles were normal, with average flow, bleeding for three to four days, and cycle duration of 27 to 30 days. Neither dysmenorrhea nor clot passage was present during her cycles. She had never experienced vaginal discharge, fever, injury, or pelvic pain. There was no relevant past medical, surgical, or therapeutic history. The bedside urine pregnancy test was positive. She was pregnant with 11 weeks' gestation and had conceived spontaneously. The patient had never taken any hormonal therapy. Since the patient was a juvenile, consent was obtained from both the patient and her parents. A medicolegal case was registered.

Upon physical examination, she was found well-nourished; her BMI was 22.1 kg/m^2^, her blood pressure was 114/72 mmHg, her pulse rate was 102 bpm (beats per minute), and she was afebrile. Pallor and oedema were absent. The air entry was bilaterally equal, with no adventitious sounds. Per abdominal examination revealed a doughy feel, and external ballottement was absent. The uterus was palpable, corresponding to 20 weeks. There was discomfort and rebound tenderness over the right iliac fossa. Examination with a speculum revealed no abnormalities. The bedside pregnancy test was positive. Table 1 summarises the findings of the investigations, which showed that the blood investigations were normal.

**Table 1:** Laboratory investigation findings β-hCG: beta-human chorionic gonadotropin.

Sr. No	Investigations	Observed value	Expected value
1	Haemoglobin	11.4	12–16
2	WBC (per cumm)	8000	4000–11,000
3	Platelets (L/cumm)	2.6	1.5–4
4	International normalised ratio (INR)	1.0	0.8–1.1
5	Prothrombin time	11.6	11.9
6	Activated partial thromboplastin time (APTT)	30	29.5
7	β-hCG (mIU/L)	69,000	11,500–289,000
8	Thyroid-stimulating hormone (mIU/L)	3.2	0.5–5.0
9	Free T3 (pg/dL)	3.6	2.3–4.1
10	Free T4 (pg/dL)	12.23	9.0–17.0
11	Albumin (g/dL)	3.9	3.5–5.0
12	Total bilirubin	0.9	0.2–1.3
13	Aspartate aminotransferase	42	<50
14	Alanine aminotransferase	30	17–59
15	Serum urea (mg/dL)	22	6–24
16	Serum creatinine (mg/dL)	1.0	0.7–1.2
17	Serum sodium (mEq/L)	140	131–145
18	Serum potassium (mmol/L)	4.0	3.6–5.2
19	Glycated haemoglobin (HBA1c)	4.0	≤5.6
20	Random blood sugar	88	70–100 mg/dL

**Figure 1:**
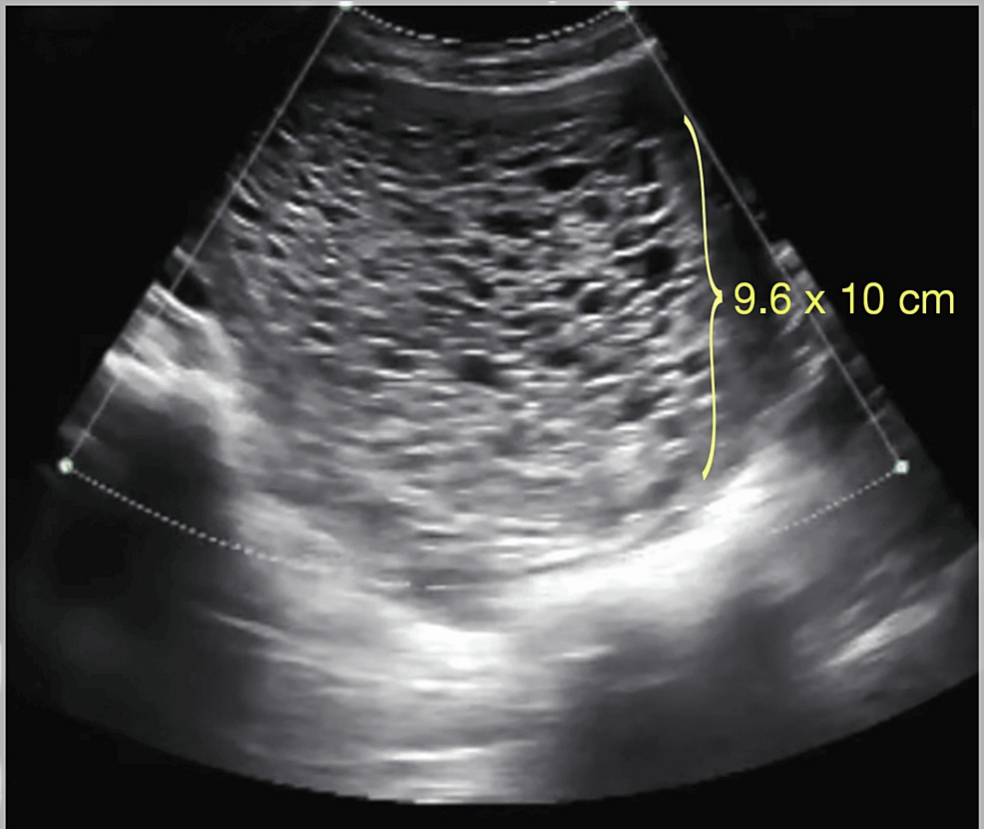
Thickened heterogeneous lesion noted in the intrauterine endometrial cavity with multiple echogenic septa with a small cystic area of size 9.6 x 10 cm noted with no evidence of obvious lesion in the adjacent myometrium.

**Figure 2:**
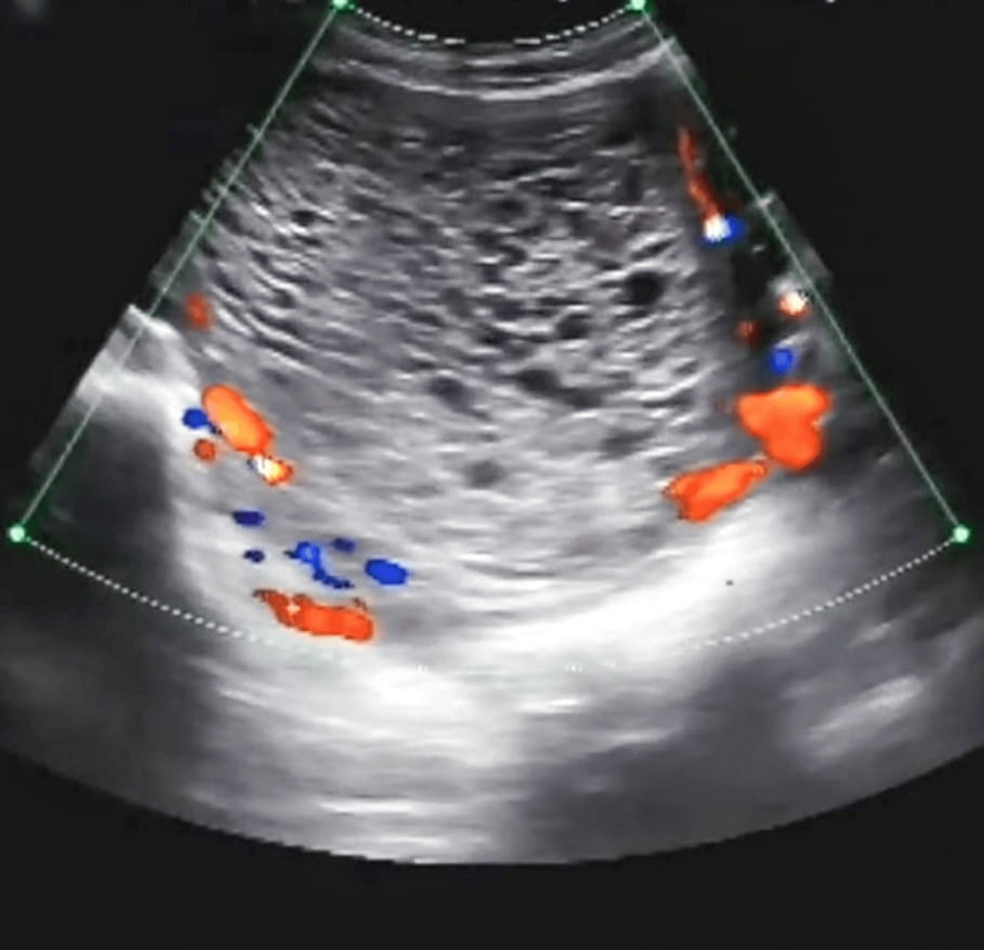
Moderate amount of peripheral vascularity and minimal internal vascularity.

Urine analysis results were within normal limits, the blood group was B-positive, and she tested seronegative for hepatitis B surface antigen and the human immunodeficiency virus. On ultrasound, a thickened heterogeneous lesion was noted in the endometrial cavity with multiple echogenic septa, and small cystic areas were noted within it, measuring 9.6 x 10 cm with a moderate amount of internal vascularity, with minimal internal vascularity, suggestive of a complete molar pregnancy (Figures 1, 2).

**Figure 3:**
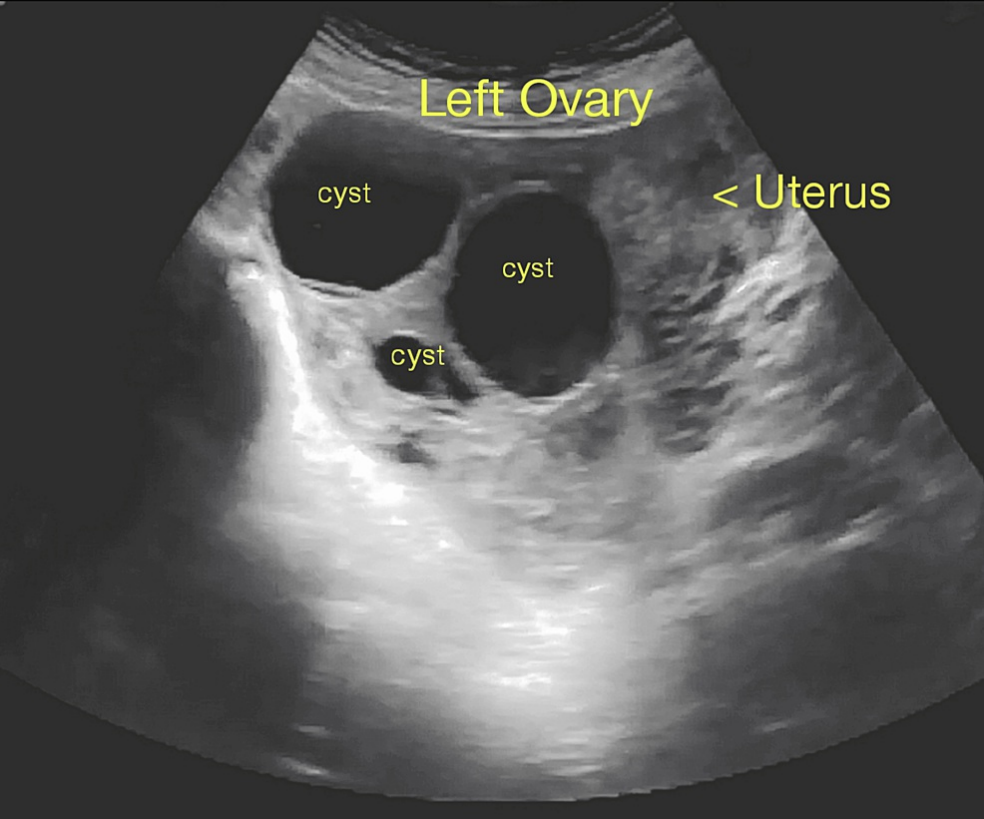
Enlarged left ovary measuring 22 cc. Multiple cystic lesions with the largest cyst measuring 36 x 48 mm.

**Figure 4:**
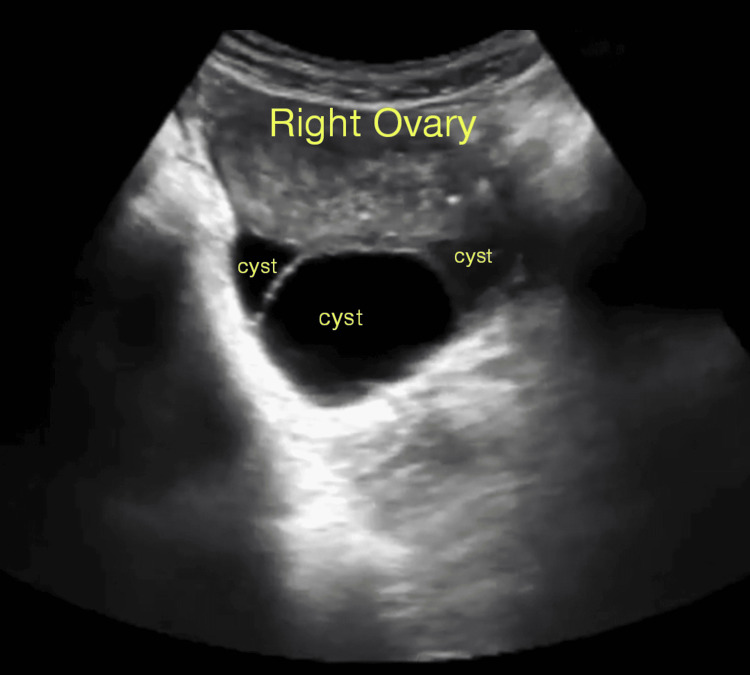
Enlarged right ovary measuring 30 cc. Multiple cystic lesions with the largest cyst measuring 34 x 52 mm.

Bilateral ovaries were enlarged, measuring 22 cc and 30 cc in the left and right ovary respectively. Multiple enlarged cysts were noted in both ovaries, with the largest cyst measuring 36 x 48 mm in the left ovary (Figure 3) and 34 x 52 mm in the right ovary (Figure 4), respectively, suggestive of TLCs with free fluid of minimal volume in the lower part of the abdomen and pouch of Douglas. After informed verbal and written consent from the parents and patient, under all aseptic precautions, evacuation was performed, revealing about 30 g of multiple grape-like vesicles. The patient tolerated the procedure well. Methotrexate (MTX: 50 mg) 1 mg/kg intramuscular (IM) on alternate days (0, 2, 4, and 6) and folinic acid (FA: 5 mg) 0.1 mg/kg IM on alternate days (1, 3, 5, and 7) were administered as part of the rescue regimen. She was also treated with antibiotics and IV fluids for eight days, and the postoperative course was uneventful. She was discharged on Day 9.

Suddenly, on postoperative day 11, she visited the Casualty Department with acute pain in the abdomen, which was sudden in onset and progressive in nature. She was afebrile, her blood pressure was 90/50 mmHg, and her pulse rate was 112 bpm with no pallor and oedema. An abdominal examination revealed a soft, tender abdomen with guarding and rebound tenderness in the right pelvic region. An emergency ultrasound was performed, revealing free fluid in the cul-de-sac. Acute appendicitis was ruled out.

**Figure 5:**
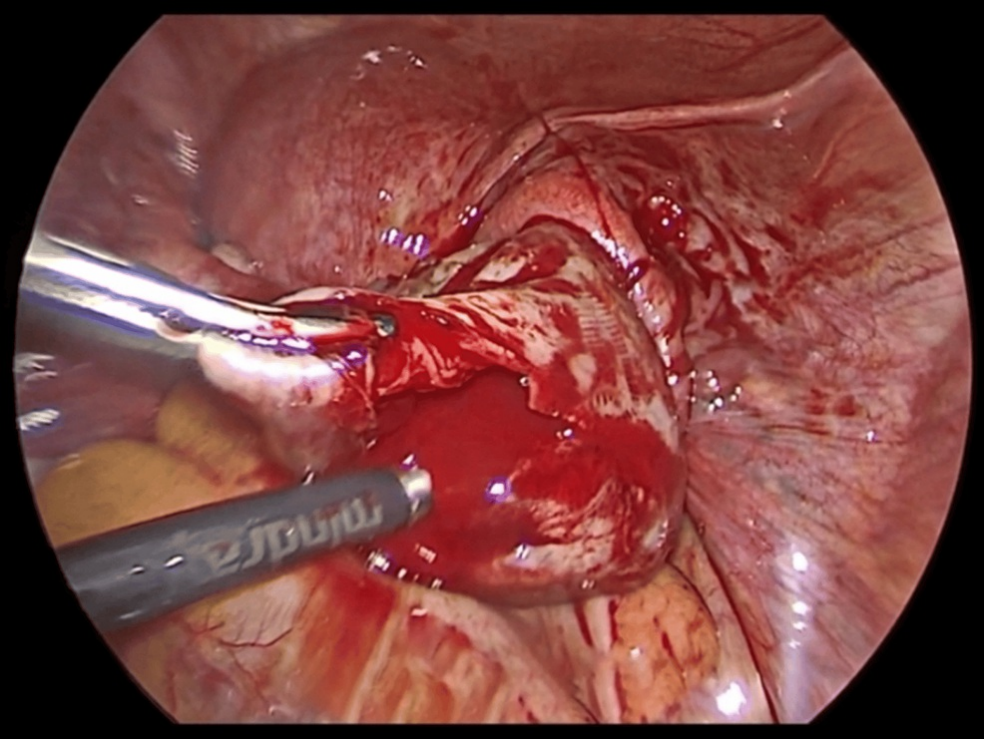
Ruptured cyst in the right ovary.

**Figure 6:**
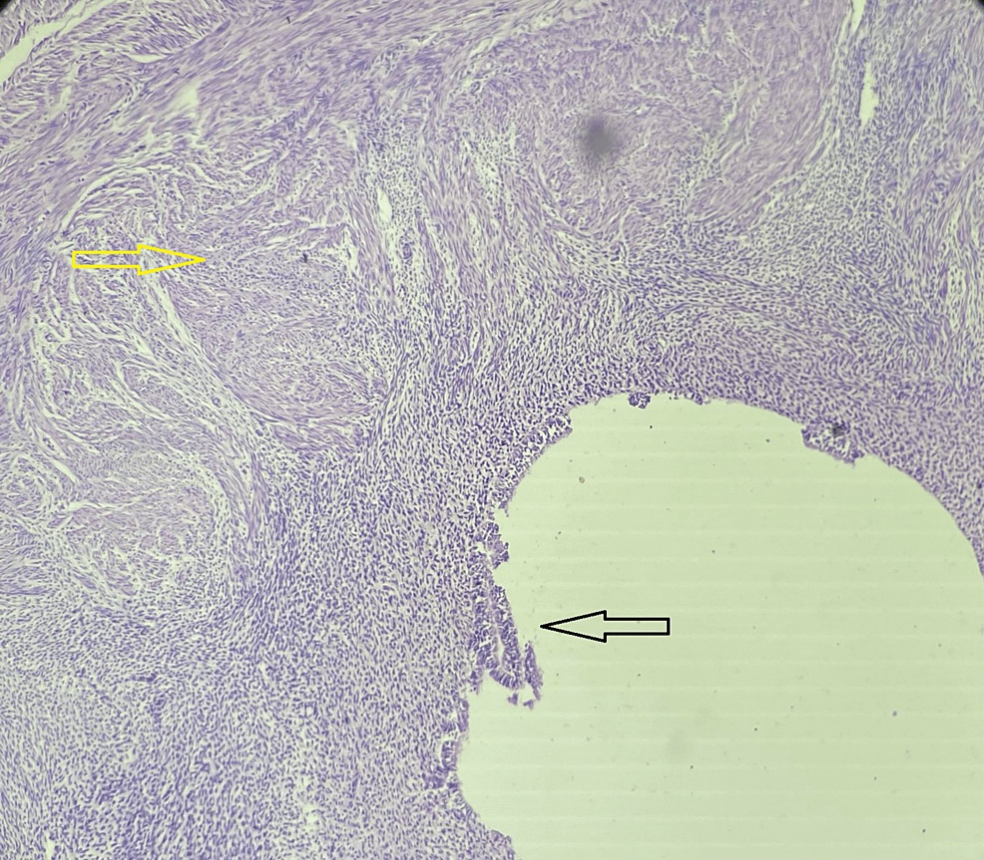
Histopathology (haematoxylin and eosin stain) shows an inner layer of luteinized polygonal shaped cells (black arrow) with abundant eosinophilic cytoplasm, granulosa cells, and the outer layer of theca cells (yellow arrow) in the fibro-collagenous stroma. Magnification: 4X.

The patient was scheduled for an emergency laparoscopy. It revealed approximately 100 cc of fresh blood in the pouch of Douglas and a ruptured cyst in the right ovary (Figure 5). Bleeding areas at the site were identified and cauterised with bipolar cautery. Hemostasis was achieved. The other ovary, fallopian tube, and the uterus were normal. The cyst wall was sent for histopathological examination. The patient was shifted to the postoperative room. Postoperatively, antibiotics were administered for seven days. The cyst wall histopathological examination revealed haematoxylin and eosin staining shows an inner layer of luteinized polygonal-shaped cells with abundant eosinophilic cytoplasm, granulosa cells, and an outer layer of theca cells in fibro collagenous stroma, suggestive of TLCs (Figure 6). She was discharged on the 7th day postoperatively. Serum β-hCG monitoring was conducted weekly and showed a declining trend (from 69,000 mIU/mL to 3000 mIU/mL in the successive week following methotrexate treatment). The histopathology examination (HPE) report from the cyst wall confirmed a TLC. She received weekly chemotherapy for eight weeks until her β-hCG level returned to the normal limit (3 mIU/mL).

## Discussion

HL, a synonym for TLCs, is caused by beta-hCG (β-hCG) hypersensitivity or by overstimulating luteinised ovarian follicle cysts due to high levels of β-hCG. This rare condition is characterised by bilateral functional ovarian cysts that are multilocular, benign, and filled with clear, straw-coloured fluid. Due to their fullness and compression, these cysts lead to ovarian enlargement and frequently result in dull, excruciating pelvic pain [3]. It is believed that excessively high amounts of circulating β-hCG cause the development of theca-lutein cysts. Bilateral theca-lutein cyst development has been reported in women with normal pregnancies, though such cases appear rare [4]. These cysts may persist even after pregnancy termination, contrary to the typical regression observed when β-hCG levels decline [5]. The association of TLCs with a partial mole is rare [2].

GTD is classified by the World Health Organization (WHO) as epithelioid trophoblastic tumours (ETT), hydatidiform moles (complete and partial), placental site trophoblastic tumours (PSTT), invasive moles, and choriocarcinoma [6]. In complete hydatidiform mole (CHM), a fetus does not develop from the fertilised egg, which contains two sets of paternal DNA instead of maternal DNA. In a partial mole, the amount of paternal DNA in the fertilised egg is double that of the mother's DNA. As a result, the embryo does not grow into a viable fetus and only partially develops. In 90% of cases, PHM is triploid with a 69 XXX karyotype, expressing both maternal and paternal DNA, whereas CHM is usually diploid with a 46 XX karyotype, expressing only paternal DNA [7]. It is uncommon for a partial mole to develop into an invasive form [1]. It has been found that 10% to 15% of complete moles and around 0.5% of partial moles develop into gestational trophoblastic neoplasia (GTN) [8]. Approximately 1-4% of pregnant women have adnexal masses [9].

In a rare case, bilateral TLCs within the initial 14 weeks of a singleton gestation resulted in acute abdominal pain (torsion of the left ovary), which required a left-side salpingo-oophorectomy and a right ovarian cystectomy [10]. In another case, a 30-year-old primigravida at 10 weeks' gestation was treated conservatively with low-molecular-weight heparin (LMWH) and albumin infusion for HL [3]. There is also a case study that describes HL after delivery, accompanied by elevated levels of β-hCG, estradiol, and testosterone. These levels spontaneously returned to normal following delivery [11]. Unfavourable pregnancy outcomes have been associated with elevated levels of human chorionic gonadotropin, including intrauterine growth restriction, preeclampsia, and poor fetal morphology [2]. Patients with a history of hydatidiform mole are more susceptible to subsequent molar pregnancies, with a 20% increased risk following the second molar conception [12].

The gold standard for diagnosis is histological analysis, while transvaginal sonography (TVS) is a useful diagnostic tool revealing a characteristic snowstorm appearance with grape-like vesicles without a clear embryonic structure [13]. Magnetic resonance imaging (MRI) aids in differentiating bilateral TLCs from ovarian malignancy, showing the characteristic "spoke wheel" appearance without solid components [6]. The nonspecific cancer antigen 125 (CA-125), which is frequently used to detect epithelial ovarian cancer, can be raised during the first trimester of pregnancy as well as the first few weeks in the postnatal period [9]. Curettage and suction, the recommended initial therapy, may lead to an unfavourable diagnosis. Chemotherapy is recommended by international standards for extrauterine disease, whereas non-conservative methods such as hysterectomy and bilateral salpingectomy are indicated for cases where there is no desire for fertility [13].

## Conclusions

A recurrent molar pregnancy is more common in patients with partial molar pregnancies. GTD is a rare phenomenon and is known to be associated with TLCs, which do not require any specific management apart from the specific care of GTD and are expected to regress spontaneously after the evacuation of the disease. In this report, the patient presented as a case of acute abdomen even after the evacuation of the hydatidiform mole; the TLCs underwent rupture before they regressed and presented as a clinical emergency. The present case adds to the knowledge of clinical practice that TLCs can create acute emergencies in the form of rupture and haemorrhage. Very rarely, it may need operative intervention. Hence, patients in the convalescence and follow-up period of GTD should be made aware of such rare events and should be encouraged to report them earlier, as and when needed.
